# A High-Throughput Cell-Based Luciferase Reporter Assay for Identifying Inhibitors of ASGR1

**DOI:** 10.3390/ijms26104590

**Published:** 2025-05-10

**Authors:** Lingmin Gan, Haoyu Zou, Zhaoqi Yang, Juntao Wang, Yunzhi Sheng, Pengfei Du, Shikun Zhang, Zili Feng

**Affiliations:** 1 School of Biological Science and Engineering, Shaanxi University of Technology, Hanzhong 723099, China; glm1632024@163.com (L.G.); 18392801420@163.com (Z.Y.); wjunntao@163.com (J.W.); syz20000416@163.com (Y.S.); 19829073668@163.com (P.D.); 2 Academy of Military Medical Sciences, Beijing 100850, China; haoyu0504@126.com

**Keywords:** firely luciferase, ASGR1, Crispr-Knockin, high-throughput drug screening

## Abstract

The asialoglycoprotein receptor 1 (ASGR1) represents a highly promising target for drug development, with its expression regulation closely linked to various diseases. Consequently, research concentrating on targeted therapies against ASGR1 holds significant importance in devising effective treatment strategies. In this study, we utilized the CRISPR-Knockin technology to insert a firely luciferase reporter gene downstream of exon 9 of the ASGR1 gene in HepG2 cell line. This modification enables the expression level of luciferase to be directly proportional to the activity intensity of the ASGR1 protein. We successfully established a drug screening and evaluation model for ASGR1 and employed it for high-throughput screening of potential inhibitors from a microbial molecular metabolite library. After our screening process, several promising candidates were identified as potential ASGR1 inhibitors. Western blotting experiment was conducted to validate the efficacy of our drug screening model, thereby providing a solid experimental foundation for the development of novel targeted therapeutics targeting ASGR1.

## 1. Introduction

The asialoglycoprotein receptor (ASGPR), also known as the hepatic galactose/N-acetylglucosamine receptor or the Ashwell–Morell receptor [1], is predominantly expressed on the surface of hepatocytes within the sinusoidal space of the liver and is recognized as one of the first identified lectins. As a member of the C-type lectin family, ASGPR exhibits calcium ion-dependent binding to its ligands. The structure of ASGPR consists of two type II single-pass transmembrane glycoprotein subunits: the major subunit ASGR1 (H1) and the minor subunit ASGR2 (H2) [2]. In humans, ASGPR has an approximate molecular weight of 46 kDa for H1 and 50 kDa for H2, with a content ratio estimated at about 2:1 [3]. The primary physiological function of ASGR1 is to facilitate the clearance of desialylated glycoproteins from circulation [4]. Within the liver, these glycoproteins are recognized and bound by ASGR1 prior to their internalization by hepatocytes through receptor-mediated endocytosis, followed by degradation in lysosomes [5]. In addition to its role in protein removal, ASGR1 also participates in various functions including cholesterol regulation, response to viral infections, and platelet regeneration.

Human genetic studies have established that variants of ASGR1 deficiency are associated with reduced cholesterol levels and a decreased risk of cardiovascular disease [6]. ASGR1 deficiency can upregulate Liver X Receptor Alpha (LXRα), ATP-binding cassette transporter A1(ABCA1), and ATP-binding cassette transporters G5 and G8 (ABCG5/G8), inhibit Sterol regulatory element-binding protein 1 (SREBP1) and lipogenesis, thereby promoting cholesterol excretion and reducing lipid levels [7]. The ASGR1 protein is also thought to play a role in facilitating infection by specific viruses, including hepatitis B [8] and SARS-CoV-2 [9]. Existing research indicates that ASGR1 likely interacts with the SARS-CoV-2 spike (S) protein through non-receptor binding domain epitopes, such as the N-terminal domain and C-terminal domain, located within the S1 region. Therefore, the suppression of ASGR1 could potentially intercept its interaction with SARS-CoV, substantially diminishing the risk of viral infection [10]. ASGR1 is also believed to be involved in the occurrence and progression of certain cancers. For example, ASGR1 is downregulated in liver hepatocellular carcinoma (LIHC) tissues as compared to non-tumorous tissues [11], and it has also been determined to be negatively correlated with the tumor progression of LIHC [12]. Besides, ASGR1 is underexpressed in lung squamous cell carcinoma and cholangiocarcinoma, while it is overexpressed in colon adenocarcinoma [13]. Targeting ASGR1 could represent an essential strategy for cancer treatment. Studies have also found that the absence of ASGR1 exacerbates liver injury, while overexpression of ASGR1 can alleviate liver damage. This is achieved through the activation of endoplasmic reticulum stress via the ASGR1-GP73 axis [14]. This indicates that ASGR1 may serve as a novel therapeutic target for the study of liver injury. Moreover, The hepatocyte Ashwell–Morell receptor interacts with Notch1 to enhance the production of thrombopoietin, with ASGR1 being a component of the hepatocyte Ashwell–Morell receptor [15]. This finding opens up new avenues for research into the role of ASGR1 in treating hematopoietic system disorders such as thrombocytopenia [16]. These research findings indicate that the downregulation of ASGR1 is associated with the treatment of various diseases. Therefore, the development of ASGR1-targeted pharmaceuticals is of profound importance.

Currently, the development of ASGR1-targeted drugs is progressing rapidly, involving various forms such as monoclonal antibodies, bispecific antibodies, siRNA, and ImmunoTAC conjugates. These drugs have shown promising therapeutic potential in both preclinical and clinical stages [17]. For example, AMG 529, a human monoclonal antibody targeting ASGR1 developed by AMGEN, demonstrated good tolerability and therapeutic potential in a Phase I clinical trial published in the Journal of the American College of Cardiology [18]. Additionally, Surrozen’s bispecific antibody SZN-043 targeting ASGR1 and Silverback Therapeutics’ ASGR1-TLR8 ImmunoTAC conjugate SBT8230 have also shown promising prospects in preclinical or early clinical stages [19,20]. Moreover, a recent study has pinpointed three potential natural inhibitors of ASGR1 through a sophisticated pharmacophore and docking-based virtual screening process [21]. However, to date, there has been a notable absence of comprehensive pharmacological profiling efforts aimed at systematically identifying and characterizing inhibitors and agonists targeting the ASGR1 receptor pathway.

To effectively and economically identify potential drug targets for ASGR1, we have developed a high-throughput screening model referred to as the ASGR1-luciferase(luc) drug screening model. By utilizing CRISPR-Knockin technology to insert a firefly luciferase reporter gene [22] sequence downstream of EXON9 in the ASGR1 gene within HepG2 cells, the expression of luc is directly proportional to the activity of the ASGR1 protein in this established system. This model enables high-throughput screening of microbial molecular metabolite libraries for drugs that target ASGR1, thereby providing a robust experimental foundation for the development of therapeutics aimed at ASGR1.

## 2. Results

### 2.1. Identification and Verification of Monoclonal Cell Lines Expressing Luc

**Figure 1:**
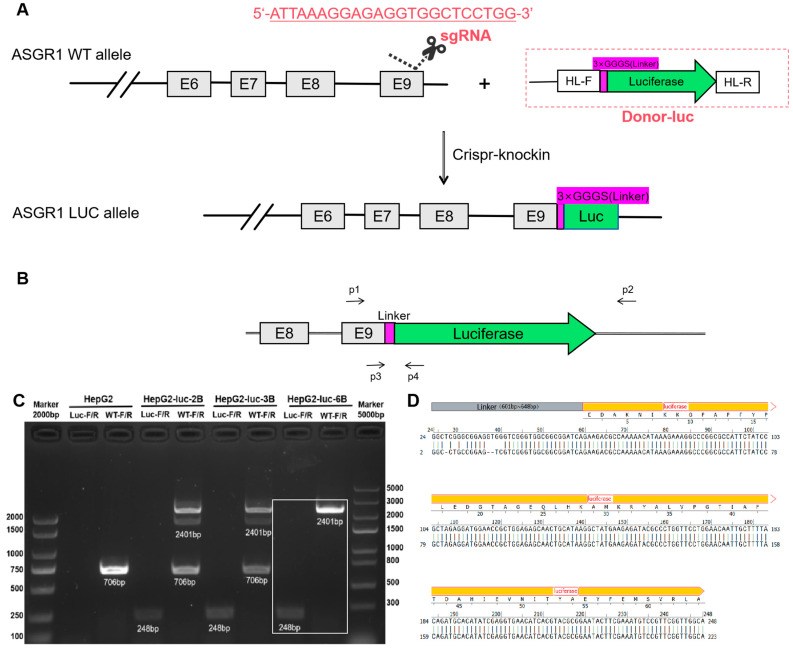
Generation and PCR identification of endogenous ASGR1-luc reporter cell line. (**A**) Schematic representation of the donor plasmid and the CRISPR/Cas9 targeting strategy. (**B**) Illustration of PCR primer amplification site. P1 and P2 represent primers WT-F and WT-R; P3 and P4 represent primers Luc-F and Luc-R. (**C**) PCR results of HepG2-luc-2B, HepG2-luc-3B, and HepG2-luc-B6; (**D**) The expected modifications of the ASGR1 locus were confirmed by Sanger sequencing (the upper base sequence represents the desired sequence, and the lower base sequence is the sequencing result of HepG2-luc-B6).

Following monoclonal screening, three HepG2-luc cell lines, namely HepG2-luc-2B, HepG2-luc-3B, and HepG2-luc-6B, which displayed relatively high luc expression values, were selected from the luc-transfected HepG2 cells for subsequent analysis. PCR was utilized to amplify the ASGR1-luc junction region for confirming the knock-in event in individual colonies with primers WT-F(p1), WT-R(p2), Luc-F(p3), and Luc-R(p4). The expected size of the PCR product for the ASGR1 WT allele and the ASGR1 luc allele was 706 base pair(bp) and 2401 bp, respectively. The results are presented in Figure 1C.

The experimental outcomes suggest that merely a 706 bp wild-type band was amplified in wild-type HepG2 cells (Figure 1C). In contrast, PCR amplification of the HepG2-luc-2B and HepG2-luc-3B cell lines produced two bands at 706 bp and 2401 bp. The result suggests that while these cell lines have successfully incorporated the luc gene sequence, they still retain the genetic background of wild-type HepG2 cells. Consequently, both HepG2-luc-2B and HepG2-luc-3B are classified as heterozygous luc expression cell lines and are deemed unsuitable for stable passage. In comparison, the HepG2-luc-6B cell line exhibited only a 2401 bp band with no detectable presence of the 706 bp band. This result indicated that this particular cell line has successfully integrated the luc gene sequence.

To ensure accuracy in our findings, we employed specific primers Luc-F and Luc-R (sequences detailed in Table 1) to perform PCR amplification on the HepG2-luc-6B cell line, resulting in a successful amplification of a target band measuring 248 bp (Figure 1C). Subsequently, Sanger sequencing was conducted on the amplified DNA fragment; sequencing results confirmed accurate insertion of the luc gene sequence as illustrated in Figure 1D. Based on these reliable experimental outcomes, we have decided to utilize the HepG2-luc-6B cell line for subsequent drug screening studies.

### 2.2. High-Throughput Drug Screening of 517 Compounds

**Figure 2:**
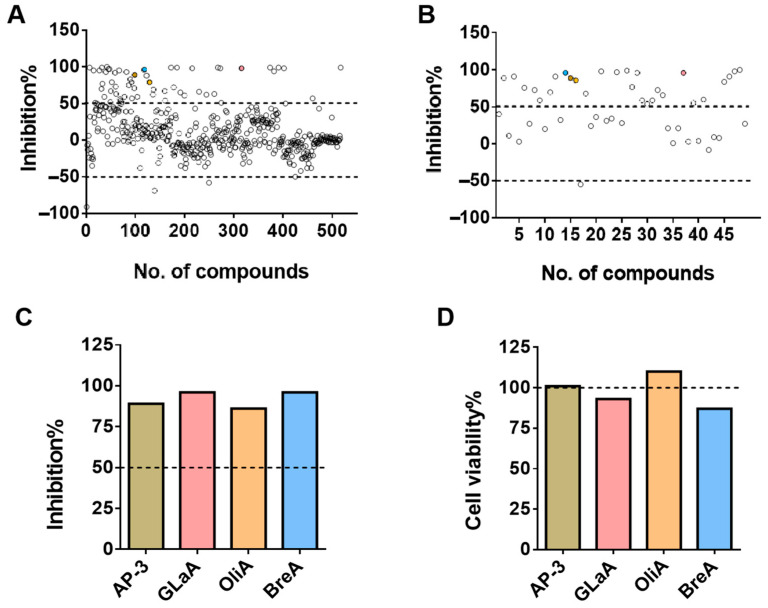
High-throughput drug screening (four points of different colors represent four selected compounds). (**A**) Results of preliminary screening of firefly luciferase inhibition activity of all compounds in the microbial library. (**B**) Results of firefly luciferase inhibition activity after secondary validation of selected compounds. (**C**) The firefly luciferase inhibition activity for the compounds AP-3, GlaA, OliA, and BreA in secondary screening. (**D**) Cell viability for AP-3, GlaA, OliA, and BreA compounds during secondary screening.

The screening of 517 compounds from MCE’s microbial metabolite molecular library was performed at an initial dosing concentration of 10 µM, with the results presented in Figure 2A. To minimize experimental error, we selected 49 compounds that exhibited an initial luc activity inhibition rate exceeding 50% and a cell viability rate greater than 80% as preliminary hit candidates. These compounds were subsequently rescreened at the same concentration using two sets of parallel values, and the outcomes are illustrated in Figure 2B.

Four drugs, namely Ansamitocin P-3 (AP-3), Glaucocalyxin A (GlaA), Oligomycin A (OliA), and Brefeldin A (BreA), were selected for further experimental validation based on the outcomes of the secondary screening. Figure 2C,D described the inhibition rates of the secondary screening for these five compounds, as well as their cell viability rates.

### 2.3. Time-Dependent Inhibitory Effects of Four Potential Inhibitors on ASGR1 Protein in HepG2 and Huh1 Cells

**Figure 3:**
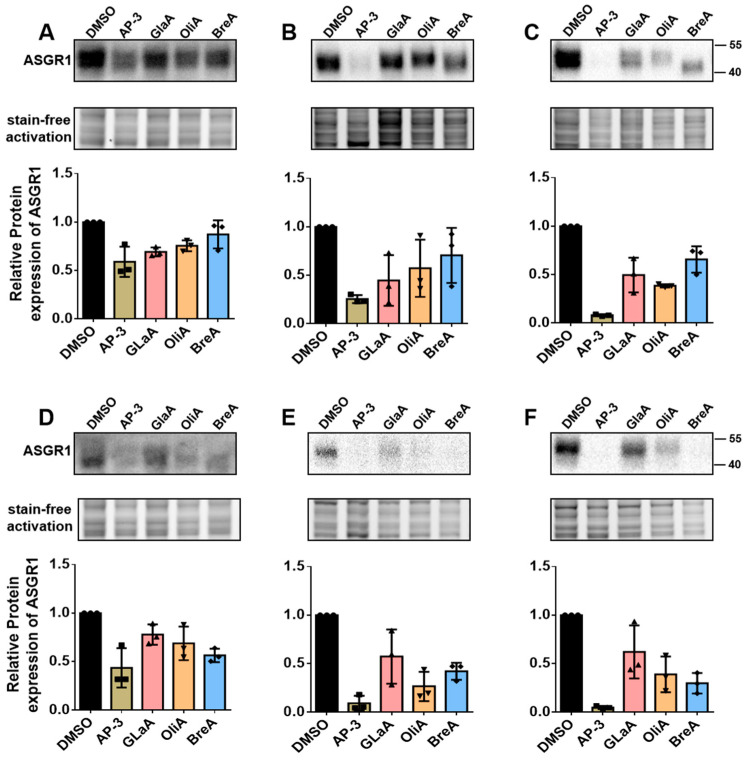
The time-dependent inhibitory effects of four inhibitors on ASGR1 protein expression in HepG2 and Huh1 cells. (**A**–**C**) show HepG2 cells were harvested for immunoblotting analysis of ASGR1 expression after being treated with 10 μM concentrations of Ap-3, GlaA, OliA, and BreA for 24, 48, and 72 h. (**D**–**F**) show the similar treatment conditions in Huh1 cells.

To ensure the reliability of the luc reporter screening results, we conducted a subsequent validation by Western Blotting in HepG2 and Huh1 cells. The four compounds that inhibit ASGR1—AP-3, GlaA, OliA, and BreA—were administered to both HepG2 and Huh1 cells at a concentration of 10 µM for 24 h, 48 h, and 72 h. The results indicated that all four inhibitors demonstrated significant inhibitory activity in both HepG2 and Huh1 cells (Figure 3), with this effect becoming more pronounced over extended exposure time. Particularly noteworthy is that among the four inhibitors tested, AP-3 demonstrated the most potent inhibitory effect, nearly completely suppressing the expression of ASGR1 protein in HepG2 and Huh1 cells after 72 h of treatment.

### 2.4. AP-3 Demonstrates a Dose-Dependent Inhibitory Effect on ASGR1 in Both HepG2 and Huh1 Cells

**Figure 4:**
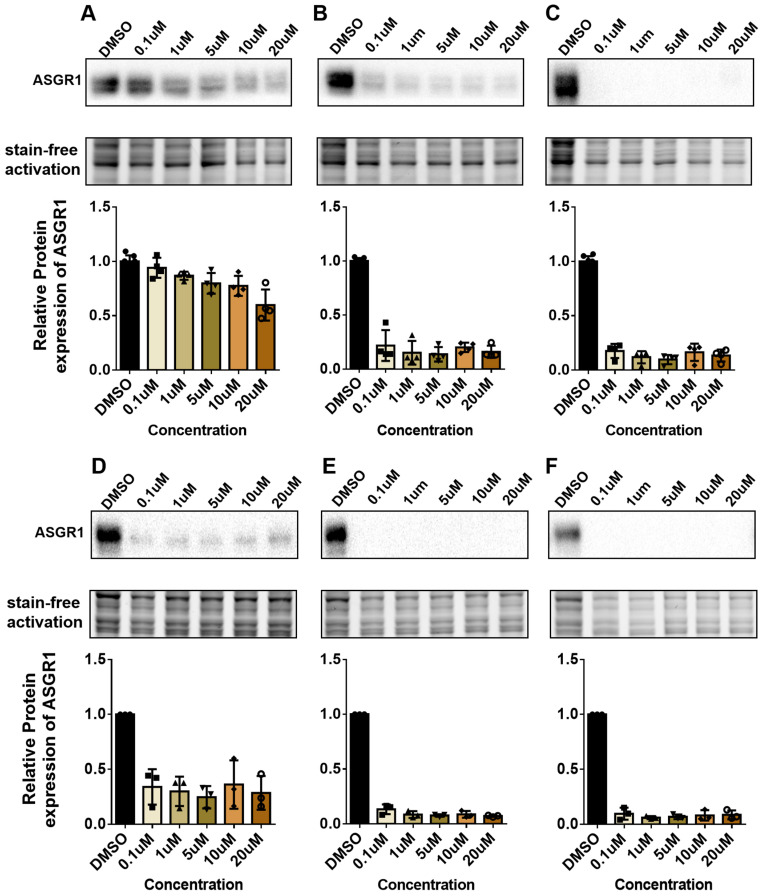
The impact of different concentrations of AP-3 on the expression levels of ASGR1 protein in HepG2 and Huh1 cell lines. (**A**–**C**) show HepG2 cells were harvested at 24 h, 48 h, and 72 h after treatment with Ap-3 at concentrations ranging from 0.1 to 20 μM for immunoblotting. (**D**–**F**) depict Huh1 cells harvested under the same treatment conditions for immunoblotting.

To comprehensively evaluate the effect of AP-3 on ASGR1 protein expression in HepG2 and Huh1 cells, we conducted a concentration inhibition experiment (Figure 4). In this experiment, we administered different concentrations of AP-3 to HepG2 and Huh1 cells and monitored the expression levels of ASGR1 protein at 24 h, 48 h, and 72 h. The results showed that under the same administration time, the inhibition rate of ASGR1 expression increased with the extension of the administration time, but this increase was not entirely positively correlated in Huh1 Cells. Specifically, at the 24-h time point, we found that a concentration of 5 µM AP-3 was most effective in inhibiting ASGR1 expression. As the treatment time was further extended to 72 h, the differences in inhibitory effects caused by different concentrations of AP-3 gradually diminished, with all tested concentrations ranging from 0.1 µM to 20 µM achieving nearly complete inhibition of ASGR1 expression.

## 3. Discussion

ASGR1 has emerged as a critical therapeutic target due to its involvement in a wide range of diseases, including metabolic disorders, viral infections, and cancer. Its regulation is closely linked to numerous pathophysiological processes, making it a promising candidate for therapeutic intervention. In this study, we developed a CRISPR-Knockin-based ASGR1-luc screening model to identify modulators of ASGR1 activity. By integrating a luc reporter gene downstream of the ASGR1 gene, we established a system that enables high-throughput quantification of ASGR1 expression. This approach successfully identified four inhibitors (Ansamitocin P-3, Glaucocalyxin A, Oligomycin A, and Brefeldin A), demonstrating the model’s sensitivity and specificity. The validation of these compounds through molecular and functional assays underscores the utility of this platform in accelerating drug discovery for ASGR1-related diseases.

The mechanisms of action of the identified modulators reveal diverse molecular pathways regulating ASGR1. AP-3 is a microtubule disruptor, while ASGR1 is a liver cell membrane receptor that highly depends on the microtubule network for endocytosis and recycling [23], so it may damage the transport and cellular localization of ASGR1. GlaA can restrain the generation of rabbit platelet-activating factor(PAF) and thromboxane A2(TXA2), eliminate the activation of target cell with PAF, maintain PGE2/TXA2 balance, and prevent artery thrombosis and atherosclerosis [24]. OliA is an inhibitor of mitochondrial ATP synthase, and the synthesis, transport, and function of ASGR1 rely on ATP energy supply [25]. OliA may interfere with the synthesis and transport of ASGR1 by inhibiting ATP production. BreA [26] may inhibit the transport and localization of ASGR1 by blocking the function of the Golgi apparatus. These findings highlight the complexity of ASGR1 regulation and its intersection with endocytosis, inflammation, and metabolic signaling, offering multiple therapeutic entry points. The functionality of this model extends beyond the discovery of ASGR1 inhibitors; it is also capable of screening for ASGR1 activators. For instance, ASGR1 inhibitors could suppress metastasis in hepatocellular carcinoma [25], whereas activators might restore metabolic homeostasis or enhance liver regeneration in specific disease contexts [14]. The therapeutic implications of these modulators extend beyond oncology. ASGR1′s role in viral interactions, such as binding SARS-CoV-2 spike protein via non-RBD epitopes [10], suggests potential antiviral applications for inhibitors. Conversely, activators could address thrombocytopenia by modulating the ASGR1-Notch1-thrombopoietin axis [15]. Current drug development efforts, including monoclonal antibodies and bispecific antibodies, further validate ASGR1′s druggability. Our screening model complements these advances by enabling rapid identification of novel modulators from microbial metabolite libraries, addressing the current lack of systematic pharmacological profiling.

Despite these advancements, certain limitations must be acknowledged. The luciferase-based system quantifies ASGR1 expression but does not directly assess its functional activity, thus necessitating complementary assays to evaluate ligand binding or downstream pathway effects. Future studies should incorporate functional validations, such as ligand uptake assays or transcriptomic analyses of pathways like LXRα and Notch1 signaling. Additionally, rigorous preclinical evaluation of in vivo pharmacokinetics and safety profiles is required. Expanding compound libraries to include natural products and synthetic analogs, coupled with computational approaches such as AI-driven pharmacophore modeling, could enhance the discovery of safer and more potent modulators.

In conclusion, this study has established a robust ASGR1-luc screening model, which effectively demonstrates the efficacy of high-throughput screening technology in the rapid identification of ASGR1 modulators. Additionally, multi-dimensional experimental validations have confirmed the accuracy and reliability of the screening results. The various identified ASGR1 inhibitors provide new candidate drugs and potential targets for the treatment of ASGR1-related diseases, laying a solid foundation for subsequent mechanistic investigations and drug development. Future research should prioritize mechanistic validation of the identified compounds and their translation into clinical applications, ultimately addressing unmet needs in oncology, metabolic disorders, and infectious diseases.

## 4. Materials and Methods

### 4.1. Cell Culture

HepG2 cells (The Cell Bank of the Chinese Academy of Sciences, Shanghai, China, SCSP-510) were cultured in MEM Medium (Gibco, Waltham, MA, USA, C11095500cp) supplemented with 10% FBS (ExcellBio, Australia, FSD500), and 1% penicillin/streptomycin (Gibco, 15140-122). Huh1 (HB-8065, ATCC, Manassas, VA, USA) cells were maintained in DMEM (Gibco, Waltham, MA, USA) supplemented with 10% FBS and 1% penicillin/streptomycin. All cell lines were maintained at 37 °C in a humidified incubator with 5% CO_2_.

### 4.2. Design and Construction of the CRISPR/Cas9 System and Donor Template to Establish ASGR1-Luc Knockin HepG2 Cells

To establish ASGR1-luc knock-in HepG2 cells, the luc reporter gene was inserted into the endogenous ASGR1 locus in HepG2 cells using CRISPR/Cas9 mediated homologous recombination.

The gene sequence of the target gene ASGR1 (Gene ID: 432) was retrieved from the NCBI Gene Bank and imported into SnapGene 7.1.2 software for analysis. Subsequently, we utilized the CRISPOR online sgRNA design tool to create sgRNA. Through an examination of the gene locus and its corresponding protein, Exon 9 in ASGR1-201 was identified as the target site for gene knock-in. Ultimately, a knock-in guide RNA (sgRNA) targeting this location was constructed within the plasmid YKO-RP003-hCAMP (sgRNA) by Ubigene Biosciences (Guangzhou, China). The sequence of the sgRNA is provided in Table 1.

The donor DNA typically consists of a linear DNA fragment with homologous arms that includes the luc gene sequence. Preceding the luc gene sequence (The luc gene sequence refer to Supplementary Materials), we designed a linker sequence (GGGS) to facilitate subsequent integration of the luc gene into the ASGR1 genomic context. (Figure 1A) The vector plasmid Donor-CAMP-3×GGGS-Luc was synthesized by Ubigene Biosciences (Guangzhou, China).

### 4.3. Generation of a Stable ASGR1-Luc Knockin HepG2 Cell Line

HepG2 cells were seeded on a 6-well plate and reached 70–80% confluence for transfection. Cells were transfected with 2.5 µg YKO-RP003-hCAMP (sgRNA) and 2.5 µg Donor-CAMP-3×GGGS-Luc donor using the Lipo3000 transfection reagent kit (Thermo Fisher Scientific, Waltham, MA, USA) in accordance with the manufacturer’s instruction. The 6-well plate was gently shaken to ensure even distribution of the transfection complex within each well. After 24 h transfection, cells were washed twice with 1 mL PBS. Cells were scraped from a six-well plate and transferred to 96-well white assay plates. To each well, 30 µL britelite™ plus (PerkinElmer, Springfield, IL, USA, 110-23171) was added, followed by incubation for 5 min at room temperature for cell lysis. The luminescence of each well was read by GloMax^®^ Navigator Microplate Luminometer (Promega, Madison, WI, USA, GM2010).

### 4.4. Preliminary Screening of HepG2-ASGR1-Luc Monoclonal Cells

After successfully introducing the luc sequence into HepG2 cells, the resulting HepG2-ASGR1-luc fluorescent cell lines were further cultured until they reached 80% to 90% confluence. Subsequently, the cells were digested using 0.25% trypsin with EDTA (Biosharp, Shenzhen, China). The cells were then seeded into 96-well plates (Corning, 3599) at a density of one cell per well. Once single-cell colonies formed in the 96-well plates, each well was gently pipetted with 15 μL of Trypsin (Biosharp, BL521A) for digestion, followed by the immediate addition of 15 μL of culture medium to terminate the digestion process.

Next, 15 μL of the cell suspension was transferred to 96-well white assay plates (PerkinElmer, 6005680), and subsequently, 20 μL of britelite™ plus was added. After thorough mixing, the cells were lysed for 5 min, and fluorescence values were measured using the Microplate Luminometer. Cells from wells exhibiting higher fluorescence values were selected and transferred to 48-well plates for expansion culture with appropriate labeling.

When the cells in the 48-well plates reached approximately 80% confluence again, they underwent another round of digestion and reseeding into new 96-well plates at a density of one cell per well for continued culture and performed the fluorescence detection. Wells displaying higher fluorescence values were chosen for further expansion culture in new 48-well plates.

Once more when cells in these newly designated wells grew to about 80% confluence again, they were reseeded into fresh 96-well plates at a density of one cell per well following similar procedures as before. This cycle continued until consistent fluorescence values among wells containing cell colonies within multiple rounds on different days became evident across all tested conditions within those same respective plate types—indicating successful clonal selection had been achieved. At this point, monoclonal cell lines could be frozen and stored for future applications.

### 4.5. Verification of Monoclonal Cell Lines

The test was performed utilizing the Monoclonal Genotype Identification Kit (No Extraction Required) (UBIGENE, Austin, TX, USA, YK-MV-100). High-fluorescence cell lines were selected alongside the control cell line HepG2. The culture medium supernatant was carefully aspirated, and the cells were washed twice with phosphate-buffered saline (PBS). A small portion of cells was scraped off using a 1 mL pipette tip and transferred to a 1 mL EP tube containing 20 μL of PBS. The mixture was then centrifuged at 1000× *g* for 2 min at room temperature. Following centrifugation, the supernatant PBS was meticulously removed, retaining the cell pellet. Subsequently, 90 μL of MicroCell DNA Lysis Buffer A was added to the cell sample, and thorough mixing was achieved by pipetting. The sample underwent heating at 95 °C for 10 min to facilitate cell lysis. After this heating step, an additional 10 μL of MicroCell DNA Lysis Buffer B was introduced into the tube to terminate the lysis process. The resulting mixture was again mixed by pipetting and stored at 4 °C in preparation for downstream PCR reactions.

The lysate products derived from the aforementioned high-fluorescence cell lines were utilized as DNA templates. PCR amplification was conducted on HepG2 cells and the selected high-fluorescence cell lines using the primers WT-F and WT-R. The sequences of these primers, along with the lengths of the amplified fragments, are presented in Table 2.

After the completion of PCR amplification, gel electrophoresis was employed to verify whether the selected cell lines were indeed monoclonal fluorescent cell lines. Given that the amplified band containing the full-length luc gene measured 2401 bp, direct sequencing was susceptible to errors. Therefore, specific primers Luc-F and Luc-R were designed to selectively amplify only the linker sequence following the ASGR1 gene and a portion of the luc sequence, resulting in a target fragment length of 248 bp, as illustrated in Figure 1. Subsequently, the target band from the gel was excised for Sanger sequencing.

### 4.6. High-Throughput Drug Screening

The selected monoclonal fluorescent cell lines were seeded at a density of 7000 cells per well into 96-well white plates (PerkinElmer, 6005680) and clear plates (Corning, Corning, NY, USA, 3599). The fluorescence value of the cells was measured on the white plates, while the cell viability was determined on the clear plates. After 24 h, the medium was replaced with 10% fresh medium, and the drug positions in the microbial metabolic molecule library were added to the corresponding cell wells at a concentration of 10 μM. A DMSO control group was also set up.

At 24 h after drug treatment, the fluorescence value of the cells on the 96-well white plate was detected using a single fluorescence detection kit; for the 96-well clear plate, after discarding the supernatant, 100 μL of 10% CCK-8 solution was added to each well, and the OD450 value of the cells in each well was detected after incubation for 1.5 h. The cell survival rate and fluorescence inhibition rate were calculated according to the formula, which is as follows:

(1) $$\mathrm{Cell}\;\mathrm{Viability}\;(\%)=\frac{A_{\mathrm{sample}}-A_{\mathrm{blank}}}{A_{\mathrm{triton}}-A_{\mathrm{blank}}}\times 100$$

(2) $$\mathrm{Inhibition}(\%)=\left(1-\frac{X_{\mathrm{sample}}}{X_{\mathrm{control}}}\right)\times 100$$

### 4.7. Western Blotting Analysis

After 24 h of drug treatment, the cells were washed twice with PBS and lysed on ice using a cell lysis buffer containing 1% broad-spectrum protease inhibitor. Following lysis, the cells were collected into a 1 mL EP tube and centrifuged at 4 °C at 12,000 r for ten minutes. The supernatant was then transferred to a new tube and mixed with 5× Loading Buffer in an appropriate ratio for subsequent use.

Subsequently, the protein extracts were separated by SDS-PAGE and transferred onto a nitrocellulose membrane. After blocking with 5% non-fat milk for two hours, the NC membrane was incubated overnight at 4 °C with ASGR1 recombinant antibody (1:5000, Proteintech, San Diego, CA, USA). Following incubation, the membrane was washed three times with 1× TBST solution for ten minutes each time. The NC membrane was then incubated at room temperature for two hours in a solution of 5% non-fat milk containing Anti-rabbit IgG HRP-linked antibody (1:5000, Cell Signaling, Danvers, MA, USA). After this incubation step, the membrane underwent another series of washes—three times with TBST for ten minutes each. Finally, chemiluminescent imaging solution (Thermo Scientific, 34578) was used to develop the membrane which was subsequently visualized using a ChemiDoc XRS+ system (BIO-RAD, Hercules, CA, USA). Image quantification was performed utilizing Image Lab 6.1.0 software (Media Cybernetics, Rockville, MD, USA).

To account for potential effects of certain drugs on internal control protein expression within cells and to ensure reliability of experimental results, total protein content normalization was conducted using stain-free internal control signals. This approach aimed to eliminate experimental errors arising from variations in total protein quantities among samples.

## 5. Conclusions

The ASGR1-luc drug screening model we developed has successfully identified four compounds—Ansamitocin P-3, Glaucocalyxin A, Oligomycin A, and Brefeldin A—all of which effectively inhibit the expression of ASGR1 protein in HepG2 and Huh1 cell lines. This achievement lays a solid foundation for subsequent high-throughput screening experiments. Among the identified inhibitors, Ansamitocin P-3 exhibits particularly pronounced inhibitory effects on ASGR1 protein in both liver cancer cell lines, with its efficacy increasing over extended treatment durations.

Furthermore, these results validate the effectiveness of our drug evaluation model. We can continue to utilize this high-throughput screening approach to identify additional ASGR1 inhibitors and activators from other compound libraries.

## Figures and Tables

**Table 1:** The sgRNA sequence targeting ASGR1 and its PAM.

Name	sgRNA Sequence (5′-3′)	PAM
sgRNA1	ATTAAAGGAGAGGTGGCTCC	TGG

**Table 2:** Primers for the identification of luc gene knockin.

Name	sgRNA Sequence (5′-3′)	Amplified Fragment Length
WT-F(P1)	TGTCCAGCACCACATAGGC	706 bp
WT-R(P2)	GCATTACGAAGCCTTAGCGG	2401 bp
Luc-F(P3)	CACCTCTCCTTTCTGGTGGC	248 bp
Luc-R(P4)	TGCCAACCGAACGGACATT	

## Data Availability

The original contributions presented in this study are included in the article. Further inquiries can be directed to the corresponding authors.

## Supplementary Materials

The following supporting information can be downloaded at https://www.mdpi.com/article/10.3390/ijms26104590/s1.
