# Supplementary material for: A High-Throughput Cell-Based Luciferase Reporter Assay for Identifying Inhibitors of ASGR1
Source: Int J Mol Sci. 2025 May 10;26(10):4590. doi: 10.3390/ijms26104590 (PMC12111582; doi:10.3390/ijms26104590)
Supplement: Supplementary file 1 [file ijms-26-04590-s001.zip › ijms-3531835-supplementary .pdf]

The vector plasmid Donor-CAMP-3×GGGS-Luc sequence:

CTGGGTGGAGCACGAGCGCAGCTGCTACTGGTTCTCTCGCTCCGGGAAGGCCTGGGCTGACGC
CGACAACTACTGCCGGCTGGAGGACGCGCACCTGGTGGTGGTCACGTCCTGGGAGGAGCAGGT
GAGGACCCGGAGGGTCTGGGAGGCTGGCTGGCCTCGGAGAGATCACCACCCGCCTTCTCTCTC
CTCAGAAATTTGTCCAGCACCACATAGGCCCTGTGAACACCTGGATGGGCCTCCACGACCAAA
ACGGGCCCTGGAAGTGGGTGGACGGGACGGACTACGAGACGGGCTTCAAGTGAGTGCGCGCCC
TCCCTCGGCCTGGGTCCGGCCGCCTTCGCGCCCTGGGGCCCTGGGCTGAGGAGTCTGGAGCGA
CCCGCCTGCGGATCCGACCTCCTGGGGCCCACAGCTGGCTCTGTCCCCAGGAACTGGAGGCCG
GAGCAGCCGGACGACTGGTACGGCCACGGGCTCGGAGGAGGCGAGGACTGTGCCCACTTCACC
GACGACGGCCGCTGGAACGACGACGTCTGCCAGAGGCCCTATCGCTGGGTCTGCGAGACAGAG
CTGGACAAGGCCAGTCAGGAGCCACCTCTCCTTTCTGGTGGCGGAGGCTCGGGCGGAGGTGGG
TCGGGTGGCGGCGGATCAGAAGACGCCAAAAACATAAAGAAAGGCCCGGCGCCATTCTATCCG
CTAGAGGATGGAACCGCTGGAGAGCAACTGCATAAGGCTATGAAGAGATACGCCCTGGTTCCT
GGAACAATTGCTTTTACAGATGCACATATCGAGGTGAACATCACGTACGCGGAATACTTCGAA
ATGTCCGTTCGGTTGGCAGAAGCTATGAAACGATATGGGCTGAATACAAATCACAGAATCGTC
GTATGCAGTGAAAACTCTCTTCAATTCTTTATGCCGGTGTTGGGCGCGTTATTTATCGGAGTT
GCAGTTGCGCCCGCGAACGACATTTATAATGAACGTGAATTGCTCAACAGTATGAACATTTCG
CAGCCTACCGTAGTGTTTGTTTCCAAAAAGGGGTTGCAAAAAATTTTGAACGTGCAAAAAAAA
TTACCAATAATCCAGAAAATTATTATCATGGATTCTAAAACGGATTACCAGGGATTTCAGTCG
ATGTACACGTTCGTCACATCTCATCTACCTCCCGGTTTTAATGAATACGATTTTGTACCAGAG
TCCTTTGATCGTGACAAAACAATTGCACTGATAATGAACTCCTCTGGATCTACTGGGTTACCT
AAGGGTGTGGCCCTTCCGCATAGAACTGCCTGCGTCAGATTCTCGCATGCCAGAGATCCTATT
TTTGGCAATCAAATCATTCCGGATACTGCGATTTTAAGTGTTGTTCCATTCCATCACGGTTTT
GGAATGTTTACTACACTCGGATATTTGATATGTGGATTTCGAGTCGTCTTAATGTATAGATTT
GAAGAAGAGCTGTTTTTACGATCCCTTCAGGATTACAAAATTCAAAGTGCGTTGCTAGTACCA
ACCCTATTTTCATTCTTCGCCAAAAGCACTCTGATTGACAAATACGATTTATCTAATTTACAC
GAAATTGCTTCTGGGGGCGCACCTCTTTCGAAAGAAGTCGGGGAAGCGGTTGCAAAACGCTTC
CATCTTCCAGGGATACGACAAGGATATGGGCTCACTGAGACTACATCAGCTATTCTGATTACA
CCCGAGGGGGATGATAAACCGGGCGCGGTCGGTAAAGTTGTTCCATTTTTTGAAGCGAAGGTT
GTGGATCTGGATACCGGGAAAACGCTGGGCGTTAATCAGAGAGGCGAATTATGTGTCAGAGGA
CCTATGATTATGTCCGGTTATGTAAACAATCCGGAAGCGACCAACGCCTTGATTGACAAGGAT

GGATGGCTACATTCTGGAGACATAGCTTACTGGGACGAAGACGAACACTTCTTCATAGTTGAC
CGCTTGAAGTCTTTAATTAAATACAAAGGATACCAGGTGGCCCCCGCTGAATTGGAGTCGATA
TTGTTACAACACCCCAACATCTTCGACGCGGGCGTGGCAGGTCTTCCCGACGATGACGCCGGT
GAACTTCCCGCCGCCGTTGTTGTTTTGGAGCACGGAAAGACGATGACGGAAAAAGAGATCGTG
GATTACGTCGCCAGTCAAGTAACAACCGCGAAAAAGTTGCGCGGAGGAGTTGTGTTTGTGGAC
GAAGTACCGAAAGGTCTTACCGGAAAACTCGACGCAAGAAAAATCAGAGAGATCCTCATAAAG
GCCAAGAAGGGCGGAAAGTCCAAATTGTAATTTATTTCTTCAATGCCTCGACCTGCCGCAGGG
GTCCGGGATTGGGAATCCGCCCATCTGGGGGCCTCTTCTGCTTTCTCGGGAATTTTCATCTAG
GATTTTAAGGGAAGGGGAAGGATAGGGTGATGTTCCGAAGGTGAGGAGCTTGAAACCCGTGGC
GCTTTCTGCAGTTTGCAGGTTATCATTGTGAACTTTTTTTTTTTAAGAGTAAAAAGAAATATA
CCTAAACCTTCTGTTAGTTGTCTGGTTATTGGGGATTCGGAAGCAGGAGTGGGCTGGTTGGCA
TTACGAAGCCTTAGCGGGTGCTGTGGCATCATGAGAACTGTGTGGGCTTTGGGCCAGAATGGC
CAGACTTTGTTATTTACAGATACGTGAGTTTGGGCAAATTATTGTTCTCTGTGTCCCAGCTGT
AAACAAGCCATCTTACTGGAGGCCATCCTACTTGGAGCAATACCCCCAGGAGGAGAACTACCC
GAATTTTTTTTTTGTAAGATGGAGTCTTGCTCTGTTGCCCAGGCTGGAATGCAATGGCACGAT
CTCAGCTCACTGCAACCTCTGCCCCCCGGGTTCAAGTGATTCTCCTGCCTCAGCCTCCCG

Among them, the 1-600 positions are the upstream homology arm sequence, the 601-648 positions are the linker sequence, the 649-2298 positions are the luc gene sequence, and the 2299-2895 positions are the downstream homology arm sequence.
